# Spatiotemporal analysis of contraceptive deserts and adolescent fertility clusters in South Asia: A spatial econometric approach to breaking intergenerational poverty cycles

**DOI:** 10.1371/journal.pgph.0005233

**Published:** 2026-06-11

**Authors:** Mohammad Safi Uddin, Md Rony Masud

**Affiliations:** 1 Graduate School of Governance Studies, Meiji University, Tokyo, Japan; 2 Graduate School of Business, Rikkyo University, Tokyo, Japan; PLOS: Public Library of Science, UNITED STATES OF AMERICA

## Abstract

‘Adolescent fertility’ and ‘teenage motherhood’ are key factors that sustain cycles of poverty across generations in South Asia. Yet there is limited research on how these outcomes are geographically clustered and how ‘contraceptive deserts’, areas with very poor access to family planning, affect nearby regions. This study explores: (i) Where are the geographic clusters of adolescent fertility and teenage motherhood? (ii) How do socioeconomic factors shape these outcomes? and (iii) Do contraceptive deserts create spillover effects in neighboring districts? We tested three hypotheses: H₁: Regions with low female literacy and high child stunting have higher adolescent fertility rates (AFR) and teenage motherhood (TMM). H₂: Internet access has a stronger negative effect on AFR than on TMM. H₃: AFR and TMM show significant spatial autocorrelation (Moran’s I > 0.4). To test these, we identified spatiotemporal clusters of AFR and TMM across South Asia and analyzed their drivers using a spatial econometric approach. Specifically, we applied a dual-outcome Spatial Durbin Model (SDM) to panel data from eight South Asian countries (1990–2023), accounting for spatial lags and spillover effects. The findings show three main results. First, female literacy strongly reduces AFR (β = -1.297, p < 0.01), while child stunting increases it (β = 0.495, p < 0.05), confirming H₁. Second, spatial analysis revealed strong autocorrelation (Moran’s I > 0.4) and spillover effects (ρ > 0, p < 0.01), confirming H₃ and the role of contraceptive deserts. Third, persistent hotspots were found in neighboring districts across national borders. However, internet access did not show the expected stronger effect on AFR compared to TMM, rejecting H₂. In conclusion, adolescent fertility and teenage motherhood in South Asia are spatially connected problems, reinforced by localized gaps in education and healthcare. National-level policies alone are insufficient to address these cross-border clusters. Instead, targeted and multi-sectoral strategies in hotspot areas, such as ‘Health Equity Zones’ that improve education, nutrition, and contraceptive access together, are needed to break the cycle of poverty.

## Introduction

### Background

Although awareness regarding family planning in South Asia has led to a reduction in birth rates, population growth is projected to persist in the forthcoming century due to population momentum, driven by the high number of adolescents and youth in this region [1–3]. Hence, adolescent fertility and teenage pregnancy have become the main public health concerns and critical social development challenges for South Asian countries [4,5].

Among South Asian countries, Bangladesh faces a serious burden of adolescent childbearing. For females ages between 15 and 19 years, adolescent birth rate remains nearly 73–74 births per 1,000 girls annually, while the rate of teenage motherhood is estimated at around 240 per 1,000 women. This difference indicates that a greater proportion of adolescent girls have already experienced pregnancy or childbirth at some stage during adolescence, rather than within a particular year [6,7]. Such high rates bring about serious negative outcomes to society, such as maternal and neonatal (newborn infants within the first 28 days of life) deaths, low schooling, and multigenerational poverty [8,9]. South Asia has only around 25% of the world’s adolescent population, but nearly 40% of adolescent pregnancies are noted within the region, which reflects a severe developmental challenge for the region [10].

Adolescent fertility is influenced by various cultural and social factors. In many developing countries, high fertility during adolescence is closely linked to early marriage and social acceptance of childbearing at a young age. On the other hand, in developed countries, teenage pregnancy is related to socioeconomic disadvantage and is normally seen outside marriage [11,12]. Across South Asia, these situations lead to early marriage, low levels of female education, malnutrition, and fewer health benefits, especially among rural and socially disadvantaged communities [13,14]. Moreover, girls who become mothers during adolescence are more likely to leave school at early age, stay outside of formal employment, and face long term economic difficulties in future [15]. Another important issue for adolescent fertility is unequal access to contraceptive services in the hard-to-reach (remote) areas, which are less visible in national statistics. Researchers call these zones as “contraceptive deserts” where access to contraceptive methods is very limited due to weak supply systems, poor infrastructure, or strong cultural barriers [16].

In most cases, these gaps are not visible in national statistics, which makes it challenging to design effective policies without an elaborate, focused and localized analysis. This study addresses the problem statement and tackles crucial challenges by applying spatial econometric methods using data from eight South Asian countries, including Afghanistan, Bangladesh, Bhutan, India, the Maldives, Nepal, Pakistan, and Sri Lanka. The research aims of this study are to identify spatial clusters, explore regional socioeconomic drivers, identify spillover effects, and develop targeted interventions to reduces adolescent fertility and teenage pregnancy.

### Objectives

Existing studies have established various causes and consequences of adolescent fertility and teenage motherhood; however, the majority compare either national-level or cross-sectional data, inherently inadequate for representing local patterns or geographic relationships [4]. For this reason, some critical factors like the influence of neighboring regions, or how surrounding areas may be affected by high-fertility districts remain fully unexplored [10]. For example, studies show that fertility rates in rural areas can be higher in some percentages than the rates in urban areas, but such patterns are not visible in aggregated national data [17,18]. In addition, almost no studies have explored adolescent fertility and teenage pregnancy together, even though they often result from similar socioeconomic conditions such as low education, poverty, and lack of access to digital and health services [14,15]. This methodological gap is addressed in this research by modeling adolescent fertility and teenage motherhood mutually using spatial econometrics, providing new insights into the geographic dimensions of adolescent’s reproductive health outcomes. Therefore, in this paper, we seek to address these issues by exploring the following key research questions: *(i) Where are the geographic clusters of adolescent fertility and teenage motherhood across South Asia? (ii) How do different socioeconomic factors like female literacy, internet access, and child stunting individually influence these two outcomes? and (iii) Do hard-to-reach areas for contraceptive services (contraceptive deserts) create spillover effects in neighboring districts or countries?*

## Methodology

### Study design and theoretical framework

This study employed a quantitative, spatial econometric research design to explore the factors influencing adolescent fertility and teenage motherhood in eight South Asian countries from 1990 to 2023. A quantitative approach was chosen to ensure clear measurement, comparison, and modeling of patterns over time and across regions. To identify both direct effects within districts and spillover effects from neighboring areas, two fundamental methods were employed. Firstly, panel regression models, both for fixed and random effects, were employed to investigate the long-term relationships between key socioeconomic predictors and reproductive outcomes. Secondly, the Spatial Durbin Model (SDM) was developed to demonstrate both spatial lags in the outcome and predictors, helping to unfold whether conditions exist in one region that impact outcomes in surrounding areas. To identify clusters of high, middle, or low adolescent fertility and motherhood, Local Indicators of Spatial Association (LISA) were also considered.

This study aimed to examine the factors influencing adolescent fertility and teenage motherhood in South Asia. Eventually, this study employed a combination of panel data analysis and spatial econometric methods, where panel models uncovered the changes in drivers within regions over time, while spatial models identified how reproductive health behaviors in one area may influence surroundings. This combined method was appropriate to respond to research questions because it took into consideration local-level variables and wider regional impacts simultaneously. Both of the end-point variables, adolescent fertility rate (AFR) and teenage motherhood (TMM), were estimated in unison because they are grounded in and frequently shaped by a comparable set of social and economic circumstances. Analyzing subnational data equipped the study to explore tertiary inequalities that might not appear in national averages. Since the study used reliable international datasets along with harmonized indicators across countries, it further supported the accuracy and fairness of the analysis. The design also focuses on how nearby areas interact with one another, following the idea that regions close together often exhibit similar social and health outcomes, a phenomenon statistically known as spatial dependence [19,20]. The research follows the Social Determinants of Health framework [21], which provides a critical explanation of the influence of education, poverty, and access to healthcare on reproductive behavior. The dataset included 272 subnational regions across South Asia. The version of Stata 19 was adopted to perform all statistical analyses, along with some widely used spatial analysis tools. This consolidated research design provided a solid foundation for identifying both structural and geographical drivers of teenage motherhood in South Asia.

### Sample and data sources

The sample includes eight South Asian countries: Afghanistan, Bangladesh, Bhutan, India, the Maldives, Nepal, Pakistan, and Sri Lanka. The analysis covers the period from 1990 to 2021, which represents the longest and most complete span of harmonized data available for all variables across these countries. This timeframe captures major economic, energy, and environmental transitions in the region. Data on the ecological footprint per capita is obtained from the Global Footprint Network and measured in global hectares per person. All explanatory and control variables are drawn from the World Bank’s World Development Indicators (WDI), ensuring consistency and comparability across countries. All continuous variables are transformed into natural logarithms, which improve interpretability and allow coefficients to be read as elasticities.

### Variable definition and measurement

In this study, two adolescent reproductive health outcomes (AFR & TMM) were measured as outcome variables. AFR is defined as the number of live births per 1,000 women aged 15–19 years in a given year. It measures the intensity of adolescent childbearing and was obtained from the World Bank’s World Development Indicators. TMM refers to the proportion of women aged 15–19 years who have already given birth or are currently pregnant with their first child. Unlike AFR, TMM captures cumulative exposure to early motherhood during adolescence. Subnational estimates were derived from harmonized DHS-based indicators. According to the Social Determinants of Health framework, Female Literacy, Child Stunting, Poverty and Internet Access were selected as exposure variables. Female Literacy was measured as the percentage of women aged 15 years and above who can read and write. Child Stunting was defined as the percentage of children under five with height-for-age below 2 SD of WHO standards. Internet Access was measured as the percentage of individuals using the internet. Poverty was measured using the poverty headcount ratio at the national poverty line. As control variables, this study considered female secondary school enrollment, health expenditure as a percentage of GDP and female political representation measured as the share of parliamentary seats held by women. Before analysis, all continuous variables were converted to natural logarithms to improve distributional properties and facilitate elasticity-based interpretation.

### Data preprocessing

This study used secondary data drawn entirely from the World Bank’s World Development Indicators (WDI) database for the years from 1990 to 2023. There were 14 important indicators of the dataset, where AFR and TMM were the main outcome variables which are demonstrated in Table 1. The variables were renamed for convenience, and alphabetic data were coded to numeric formats for the sake of analysis. The data were structured as panel data according to country and year to facilitate tracking changes over time. Interpolation and imputation methods were applied to handle the missing values when gaps were small, and to prevent bias, extreme outliers in AFR were adjusted. However, units with abnormal missing information were excluded as per established guidelines to sustain data quality. Spatial boundaries were aligned using international geographic codes to comply with regional comparisons.

**Table 1:** Variables and Measurement.

Variable	Description	Source	Unit
**Dependent Variables**			
Adolescent Fertility Rate	Births per 1,000 women aged 15–19	WDI	per 1,000 women
Teenage Mothers	Numbers of teenage mothers per 1,000 women aged 15–19 who have had children or are currently pregnant	WDI	per 1,000 women
**Independent Variables**			
**Key Determinants**			
Female Secondary Enrollment	Gross secondary enrollment, female	WDI	% of gross
Poverty Headcount	% living below $4.20/day (2021 PPP)	WDI	% of population
Child Stunting	Height-for-age prevalence in children under 5	WDI	Percentage (%)
Female Labor Force	Female labor force participation rate (ages 15+)	WDI	% of female population
**Health System Indicators**			
Health Expenditure (% GDP)	Current health expenditure as % of GDP	WDI	% of GDP
Health Expenditure (per cap)	Current health expenditure per capita	WDI	Current US$
Physicians Density	Physicians per 1,000 people	WDI	per 1,000 people
**Social & Digital Factors**			
Female Literacy	Literacy rate among females aged 15+	WDI	Percentage (%)
Female Parliamentary Seats	Proportion of seats held by women in national parliaments	WDI	Percentage (%)
Urban Growth	Annual urban population growth	WDI	Annual % change
Internet Usage	Individuals using the Internet	WDI	% of population
Female Unemployment	Female unemployment rate (% of female labor force)	WDI	Percentage (%)

### Data sources and variable construction

This study is subject to certain data-related limitations. Due to incomplete annual coverage in the WDI database, some country-year observations were missing for selected indicators. To maintain continuity in balanced panel framework and reduce loss of observations, missing values were supplemented using linear interpolation based on adjacent years and, where necessary, trend-consistent extrapolation from nearest available observations. Such procedures are common in macro-panel research, though reconstructed values may not perfectly reflect actual historical conditions and may introduce some measurement bias. In addition, interpolation may smooth short-term fluctuations and partially conceal year-to-year shocks or policy effects. Since missing observations in WDI datasets are often more common in countries with weaker statistical reporting, some caution is warranted when interpreting findings, particularly for variables with relatively larger shares of imputed data.

### Rationale of choosing methods

Past studies that focused on determining the predictors of adolescent fertility or teenage pregnancy using national datasets, such as Demographic and Health Surveys (DHS), and they demonstrated strong correlations between female education, household wealth index, and teenage pregnancy [13]. For instance, women with secondary education are significantly less likely to become teenage mothers, while women who belong to families with a lower wealth index are more likely to become pregnant early [4]. However, most of these studies applied ordinary least squares (OLS) or logistic regressions, where spatial dependence was mostly unexplored. It means that outcomes in one area do not influence those in another, which may not be held in the context of South Asia’s congested geographic and social spaces.

By using panel data at the subnational level, a growing body of studies has been conducted that have allowed for time-trend analysis and disclosed country-specific effects [15–18,22]. These analyses have explored the effects of variables like education, internet access, household location, religion, child stunting and female labor participation on reproductive health outcomes over time [23–27]. Although these models are robust and efficient, they hardly consider spatial dependence and eventually disregard the impact that outcomes in one region may have significant influence on those in nearby areas, which devalues their efficacy for spatially targeted national policy planning.

Numerous studies that belong to regions like Africa, Nigeria and Brazil have shown the significance of spatial autocorrelation and spillover effects on health outcomes, employing methods including Moran’s I, Local Indicators of Spatial Association (LISA), and the Spatial Durbin Model (SDM) [28]. They have revealed that due to geographic proximity, shared infrastructure, or cultural ties, neighboring areas frequently share similar reproductive health behaviors. However, for South Asia, such analysis hasn’t been conducted yet, nor have these models been applied to study adolescent fertility and teenage motherhood simultaneously, leaving a significant gap in both theory and practice. This study hence utilizes a dual-outcome analytical framework, a combination of panel data analysis and spatial econometric methods and jointly examines patterns of adolescent fertility and teenage motherhood throughout South Asian countries to address this methodological limitation.

Derived from the research questions and addressing identified gaps, the central hypothesis of this paper stands for *H*₁*: Regions with low women literacy (< 45%) and high child stunting (> 30%) are more likely to have high Adolescent Fertility Rates (AFR) and Teenage Motherhood Moran (TMM) rates (β < -1.2; p < .01). H*₂*: Internet access has a stronger effect on reducing adolescent fertility than teenage motherhood, and H*₃*: There is significant spatial autocorrelation in AFR and TMM (Moran’s I > 0.4), implying that outcomes in one district are statistically related to those in neighboring regions.*

### Statistical methods

The primary objective of this study was to understand and summarize the dataset to generate a basic idea of the variables under study. The descriptive statistics here, including mean, median, standard deviation, and range, were calculated for all variables on the full sample and country by country and are represented in Tables 2 and 3 respectively. This procedure served to reveal general patterns and country-level exceptions in headline variables such as adolescent fertility rates, female literacy, and poverty.

**Table 2:** Summary Statistics for South Asian Countries (1990-2023).

Variable	Mean	Median	SD	Min	Max	Observations
**Adolescent Fertility (per 1,000 women aged 15–19)**	72.14	69.60	47.29	5.43	178.31	272
**Teenage Mothers (per 1,000 women aged 15–19 who have had children or are currently pregnant)**	131.81	121.00	96.89	16.00	356.00	204
**Female Secondary Enrollment (%)**	53.09	48.77	27.27	6.40	99.42	272
**Poverty Headcount (%)**	9.19e	416.00	2.49e	0.00	9.09e	238
**Child Stunting (%)**	38.16	39.40	15.19	7.40	73.60	272
**Female Labor Force (%)**	31.25	29.21	13.26	5.16	65.62	272
**Health Expenditure (% of GDP)**	4.96	3.92	3.32	1.77	23.09	272
**Health Expenditure per capita (US$)**	104.97	40.46	199.94	8.10	1150.69	272
**Physicians per 1,000 people**	0.61	0.51	0.49	0.04	2.16	272
**Female Literacy (%)**	52.85	45.50	27.48	17.00	99.00	272
**Female Parliament (%)**	13.09	9.09	9.42	2.00	33.16	272
**Urban Growth (%)**	3.32	3.13	1.65	-2.79	12.22	272
**Internet Usage (%)**	13.28	3.87	20.39	0.00	88.40	272
**Female Unemployment (%)**	7.45	7.42	4.97	0.25	26.74	272

**Table 3:** Country-Level Comparative Statistics (1990-2023).

Variable	Afghanistan	Bangladesh	Bhutan	India	Maldives	Nepal	Pakistan	Sri Lanka
**Adolescent Fertility Rate** (per 1,000 women aged 15–19)	117.1 (120.3) [34.7]	123.5 (125.5) [33.8]	43.3 (41.1) [28.3]	71.2 (55.0) [48.7]	33.4 (16.5) [35.6]	100.2 (92.3) [25.1]	65.4 (58.4) [19.5]	23.1 (22.0) [5.5]
**Teenage Mothers** (per 1,000 women aged 15–19 who have had children or are currently pregnant)	121.0 (121.0) [0.0]	273.0 (308.0) [103.9]		104.2 (79.0) [77.4]	18.9 (21.0) [2.5]	184.0 (176.0) [38.2]	89.8 (91.0) [17.9]	– –
**Female Secondary Enrollment** (%)	23.8 (15.4) [13.5]	54.7 (51.6) [13.6]	54.7 (47.5) [31.3]	55.2 (53.2) [17.3]	68.6 (76.2) [13.0]	49.7 (40.5) [22.7]	24.3 (24.8) [8.0]	93.6 (93.9) [3.1]
**Poverty Headcount** (% below $4.20/day)		1.02e (521.0) [2.50e+15]	235.6 (268.0) [169.0]	9.72e (745.0) [2.38e+15]	209.4 (259.0) [183.5]	1.74e + 15 (441.0) [3.18e+15]	2.70e (559.0) [3.99e+15]	224.3 (201.0) [140.9]
**Child Stunting** (% under 5)	47.0 (44.6) [6.8]	46.9 (44.2) [14.4]	32.1 (34.9) [12.5]	47.2 (47.8) [8.2]	23.6 (19.0) [10.1]	49.1 (49.1) [13.7]	41.9 (41.4) [4.0]	17.6 (18.1) [6.1]
**Female Labor Force** (%)	15.3 (15.2) [3.2]	31.9 (30.0) [5.6]	59.1 (58.4) [4.0]	30.4 (30.5) [3.0]	35.2 (37.0) [6.1]	23.8 (23.1) [2.7]	18.9 (19.1) [4.1]	35.4 (35.3) [2.9]
**Female Literacy** (%)	18.1 (17.0) [2.8]	46.2 (42.4) [18.0]	47.3 (45.0) [9.9]	48.7 (51.0) [11.6]	97.6 (98.0) [1.3]	37.0 (35.0) [16.8]	37.9 (39.0) [6.7]	90.0 (89.0) [1.2]
**Physicians per 1,000**	0.20 (0.21) [0.07]	0.37 (0.32) [0.16]	0.26 (0.23) [0.14]	1.13 (1.22) [0.20]	1.22 (1.37) [0.70]	0.32 (0.21) [0.31]	0.74 (0.71) [0.20]	0.62 (0.54) [0.33]
**Health Exp. (per capita)** (US$)	39.8 (30.8) [24.3]	24.6 (14.8) [20.1]	66.5 (55.9) [37.6]	38.3 (32.5) [20.6]	531.6 (441.1) [323.7]	27.8 (15.6) [23.6]	24.3 (23.4) [9.9]	86.9 (67.3) [46.8]
**Female Parliament** (%)	27.4 (27.4) [0.2]	13.7 (14.9) [6.4]	7.7 (8.5) [5.0]	10.0 (9.0) [2.6]	6.6 (6.3) [2.1]	17.8 (11.6) [13.7]	16.1 (20.6) [8.4]	5.3 (5.3) [0.4]
**Urban Growth** (%)	4.4 (4.2) [2.1]	3.5 (3.5) [0.4]	4.0 (3.4) [1.8]	2.6 (2.6) [0.3]	4.0 (4.3) [1.1]	4.1 (3.3) [1.8]	3.0 (3.1) [0.6]	1.0 (1.1) [0.8]
**Internet Usage** (%)	5.1 (1.9) [6.6]	9.1 (1.4) [14.0]	22.0 (5.2) [30.4]	11.5 (3.4) [17.0]	28.2 (13.7) [30.9]	11.3 (1.3) [16.7]	7.5 (6.7) [7.6]	11.5 (3.2) [15.3]
**Female Unemployment** (%)	12.2 (10.4) [4.1]	4.9 (5.1) [2.0]	3.3 (3.3) [1.7]	7.4 (7.7) [0.9]	5.6 (4.2) [3.9]	12.3 (12.2) [0.4]	2.1 (0.3) [2.7]	11.8 (9.7) [5.5]
**Health Expenditure (% GDP)**	11.2 (9.4) [4.0]	2.1 (2.1) [0.2]	3.8 (4.0) [0.5]	3.7 (3.7) [0.4]	8.3 (7.9) [1.4]	4.1 (3.9) [1.0]	2.5 (2.5) [0.2]	3.9 (4.0) [0.3]
**Observations**	34	34	34	34	34	34	34	34

Means (Medians) with Standard Deviations in Brackets.

**Fig 1:**
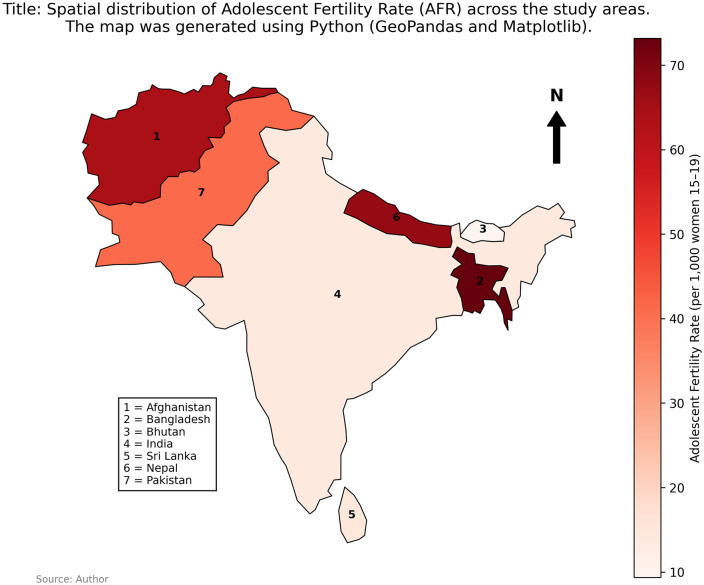
Spatial distribution of Adolescent Fertility Rate (AFR) across the study areas. The map was generated using Python (GeoPandas and Matplotlib).

After that, temporal trend analyses were conducted by plotting key indicators across the study period to measure probable changes over time. These graphical representations illustrated the ways adolescent fertility rates and teenage mother rates emerged from 1990 to 2023 in the study areas (Fig 1). Simultaneously, a correlation analysis was conducted to ascertain the relationships between all variables used in the study. The correlation matrix, shown in Table 4, revealed significant positive and negative associations among variables such as female literacy with adolescent fertility rates and poverty with adolescent fertility rates, providing negative and positive relations consecutively.

**Table 4:** Correlation Matrix with Significance Levels (Lower Triangle).

Variable (Unit)	(1)	(2)	(3)	(4)	(5)	(6)	(7)	(8)	(9)	(10)	(11)	(12)	(13)	(14)
**(1) Adolescent Fertility Rate** (per 1,000 women aged 15–19)	1.00													
**(2) Teenage Mothers (per 1,000 women aged 15–19 who have had children or are currently pregnant)**	0.56***	1.00												
**(3) Female Secondary Enrollment (%)**	-0.70***	-0.10	1.00											
**(4) Poverty Headcount (%)**	0.31***	0.13*	-0.37***	1.00										
**(5) Child Stunting (%)**	0.84***	0.41***	-0.78***	0.34***	1.00									
**(6) Female Labor Force (%)**	-0.48***	0.00	0.45***	-0.37***	-0.46***	1.00								
**(7) Health Expenditure (% of GDP)**	-0.01	-0.35***	-0.10	-0.23***	-0.10	-0.25***	1.00							
**(8) Health Exp. per capita (US$)**	-0.45***	-0.45***	0.36***	-0.17***	-0.51***	0.21***	0.35***	1.00						
**(9) Physicians per 1,000**	-0.49***	-0.46***	0.37***	-0.15*	-0.42***	0.08	0.04	0.66***	1.00					
**(10) Female Literacy (%)**	-0.72***	-0.41***	0.80***	-0.39***	-0.80***	0.40***	-0.00	0.59***	0.52***	1.00				
**(11) Female Parliament (%)**	0.21***	0.06	-0.21***	-0.21***	0.12*	-0.43***	0.34***	-0.22***	-0.14*	-0.37***	1.00			
**(12) Urban Growth (%)**	0.39***	0.08	-0.51***	0.20***	0.40***	-0.05	0.21***	0.05	-0.22***	-0.41***	0.02	1.00		
**(13) Internet Usage (%)**	-0.56***	-0.27*	0.56***	-0.24***	-0.60***	0.32***	0.11*	0.60***	0.51***	0.44***	0.08	-0.18***	1.00	
**(14) Female Unemployment (%)**	-0.01	0.13*	0.2323***	-0.1388*	-0.0478	-0.2291***	0.4493***	-0.0569	-0.1050*	0.01	0.26***	-0.07	-0.05	1.00

Significance levels: *** p<0.01, ** p<0.05, * p<0.10; n = 256 observations.

Moreover, this matrix provided valuable insight to rank potential features based on the strength of the relationship between each other for inclusion in regression models to avoid omitted variable bias. This investigation employed a rigorous two-tiered econometric framework to identify the determinants influencing the adolescent fertility rate (AFR) (Table 5) and teenage motherhood (TMM) (Table 6) within subnational administrative units. The analytical strategy effectively combined regression techniques for panel data with advanced spatial econometric modeling, addressing both temporal dynamics and spatial interdependencies.

**Table 5:** Panel regression results: core socioeconomic drivers & integrated health-labor determinants of adolescent fertility.

Variable	Model 01_01 (FE)	Model 01_01 (RE)	Model 01_02 (FE)	Model 01_02 (RE)
**Female Literacy (%)**	-1.196*** (0.188)	-1.140*** (0.169)	-1.297*** (0.193)	-0.299 (0.193)
**Female Sec. Enrollment (%)**	-0.628*** (0.129)	-0.007 (0.143)	-0.596*** (0.129)	0.441*** (0.153)
**Poverty Headcount (%)**	-7.36e (5.59e)	2.26e (8.86e)	-2.10e (4.81e)	-4.43e (7.73e)
**Internet Usage (%)**	-0.183** (0.081)	-0.654*** (0.116)	0.204** (0.100)	-0.316** (0.136)
**Health Exp. (% GDP)**	3.782** (1.627)	2.876** (1.462)	7.271*** (1.468)	-0.192 (1.542)
**Female Parliament (%)**	-0.081 (0.264)	-0.009 (0.261)	-0.354 (0.235)	0.094 (0.241)
**Child Stunting (%)**	–	–	0.495** (0.210)	2.212*** (0.227)
**Female Labor Force (%)**	–	–	-0.813** (0.326)	-0.519*** (0.179)
**Physicians per 1,000**	–	–	5.825 (4.725)	-17.749*** (4.454)
**Health Exp. per capita (US$)**	–	–	-0.068*** (0.014)	0.042** (0.017)
**Urban Growth (%)**	–	–	-4.848*** (1.032)	1.012 (1.450)
**Female Unemployment (%)**	–	–	-0.024 (0.395)	-0.861** (0.433)
**Constant**	159.007*** (9.685)	129.627*** (7.807)	174.780*** (22.007)	7.959 (20.463)
**Model Diagnostics**				
**Observations**	238	238	238	238
**Number of Countries**	7	7	7	7
**R² (Within)**	0.718	0.653	0.807	0.623
**R² (Between)**	0.421	0.519	0.466	0.885
**R² (Overall)**	0.528	0.577	0.563	0.758
**F/Wald χ²**	F(6,225)=95.65	χ²(5)=N/A	F(12,219)=76.45	χ²(11)=N/A
σᵤ **(Panel SD)**	30.654	0	34.288	0
σₑ **(Error SD)**	16.545	16.545	13.872	13.872
**ρ (Panel Variance Share)**	0.774	0	0.859	0
**Specification Tests**				
**Hausman Test χ² (p-value)**	62.06 (0.000)		144.64 (0.000)	
**Breusch-Pagan LM Test (p-value)**	0.00 (1.000)		0.00 (1.000)	

**Significance levels:** *** p < 0.01, ** p < 0.05, * p < 0.10; Coefficient value (Standard error)

**Table 6:** Panel Regression Results: Core Socioeconomic Drivers & Integrated Health-Labor Determinants of Teenage Mothers.

Variable	Model 02_01 (FE)	Model 02_01 (RE)	Model 02_02 (FE)	Model 02_02 (RE)
**Female Literacy (%)**	0.406 (0.765)	-2.068*** (0.530)	-1.691* (1.018)	-3.378*** (0.781)
**Female Sec. Enrollment (%)**	-0.923 (0.702)	2.735*** (0.506)	-1.624** (0.798)	0.644 (0.581)
**Poverty Headcount (%)**	1.07e (2.05e)	-8.09e (2.60e)	1.12e (2.08e)	8.62e (2.22e)
**Internet Usage (%)**	0.009 (0.367)	-1.012** (0.430)	0.293 (0.674)	-0.565 (0.542)
**Health Exp. (% GDP)**	4.096 (6.226)	-17.073*** (4.661)	-0.936 (6.236)	-13.041** (5.336)
**Female Parliament (%)**	-0.914 (1.014)	-0.953 (0.837)	-1.038 (1.116)	-0.431 (0.930)
**Child Stunting (%)**	–	–	-3.259*** (1.032)	-2.751*** (0.953)
**Female Labor Force (%)**	–	–	1.894 (1.879)	9.579*** (1.197)
**Physicians per 1,000**	–	–	-16.176 (27.475)	-119.964*** (15.794)
**Health Exp. per capita (US$)**	–	–	-0.077 (0.074)	0.057 (0.054)
**Urban Growth (%)**	–	–	-2.296 (6.566)	1.241 (6.252)
**Female Unemployment (%)**	–	–	4.741* (2.965)	2.025 (1.920)
**Constant**	152.130*** (33.982)	204.285*** (25.244)	400.734*** (107.994)	260.720*** (80.806)
**Model Diagnostics**				
**Observations**	170	170	170	170
**Number of Countries**	5	5	5	5
**R² (Within)**	0.071	0.000	0.194	0.104
**R² (Between)**	0.833	0.715	0.251	0.988
**R² (Overall)**	0.150	0.426	0.232	0.674
**F/Wald χ²**	F(6,159)=2.01	χ²(5)=N/A	F(12,153)=3.07	χ²(11)=N/A
σᵤ **(Panel SD)**	112.006	0	84.446	0
σₑ **(Error SD)**	59.851	59.851	56.819	56.819
**ρ (Panel Variance Share)**	0.778	0	0.688	0
**Specification Tests**				
**Hausman Test χ² (p-value)**	77.91 (0.000)		31.76 (0.000)	
**Breusch-Pagan LM Test (p-value)**	0.00 (1.000)		0.00 (1.000)	

**Significance levels:** *** p < 0.01, ** p < 0.05, * p < 0.10; Coefficient value (Standard error).

To examine sectoral effects and their interactions, three nested panel regression models are estimated. All models are estimated using both Fixed Effects (FE) and Random Effects (RE) estimators. Country fixed effects control for time-invariant characteristics such as geography and historical land-use patterns, while year fixed effects capture global shocks and common time trends. The Hausman test is used to identify the preferred specification. All estimations are conducted using two-way fixed effects, controlling for both country and year effects.

The first analytical layer relied on longitudinal panel data methods to estimate the effects of time-varying explanatory variables on AFR and TMM between 2010 and 2020. Both Fixed Effects (FE) and Random Effects (RE) models were estimated to account for unobserved heterogeneity across administrative units. The FE model assumes that each entity possesses unique characteristics that may correlate with the independent variables, and it is formally specified as:


$$\begin{array}{c} y_{it}=\alpha +X_{it}\beta +u_{i}+\epsilon_{it} \end{array}$$

where *y*_*it*_ represents the dependent variable for region α_*i*_ in year *t*, α denotes region-specific effects which are constant, *X*_*i*t_ includes observed explanatory variables (e.g., poverty rate, female literacy, health spending, internet access, female political participation), *β* is the vector of coefficients, *u*_*i*_ is **an** unobserved individual-specific effect, which captures all time-invariant characteristics of unit *i* that are not included in *X*_*it*_ and ***ϵ***_*it*_ captures random disturbances. In this paper, we used the FE models of two dependent variables following this model:


$$\begin{array}{cc} Model\;\text{1}:\;Adolescent\;Fertility_{it}=\; & \beta_{\text{0}}+\beta_{\text{1}}Female\;Literacy_{it}+\beta_{\text{2}}Secondary\;Enrollment\;Female_{it}\\ & \;\;+\beta_{\text{3}}Poverty\;Headcount_{it}+\beta_{\text{4}}Internet\;Use_{it}\\ & \;\;+\beta_{\text{5}}Health\;Expenditure\;GDP_{it}+\beta_{\text{6}}Female\;Parliament_{it}+\alpha_{i}+\varepsilon_{it} \end{array}$$

$$\begin{array}{cc} Model\;\text{2}:\;Adolescent\;Fertility_{it}=\; & \beta_{\text{0}}+\beta_{\text{1}}Female\;Literacy_{it}+\beta_{\text{2}}Secondary\;Enrollment\;Female_{it}\\ & \;\;+\beta_{\text{3}}Poverty\;Headcount_{it}+\beta_{\text{4}}Internet\;Use_{it}\\ & \;\;+\beta_{\text{5}}Health\;Expenditure\;GDP_{it}+\beta_{\text{6}}Female\;Parliament_{it}+u_{i}+\varepsilon_{it} \end{array}$$

Alongside the country fixed-effects (FE) estimates, we also estimated random-effects (RE) models to assess robustness and to exploit both within- and between-country variation. Whereas FE absorbs all time-invariant country heterogeneity through unit-specific intercepts, RE treats the unobserved country component as a random draw uncorrelated with the regressors. Formally, the RE model is


$$\begin{array}{c} y_{it}=\alpha +X_{it}\beta +u_{i}+\epsilon_{it} \end{array}$$

Interpretation under RE leverages both within and between the country information. For example, a negative coefficient on Female Literacy indicates that countries with higher female literacy tend to exhibit lower adolescent motherhood rates (between effect), and that increases in female literacy within a country over time are associated with reductions in adolescent motherhood (within effect), provided that the RE orthogonality condition holds. In this paper, we used the RE models of two dependent variables, following these models:


$$\begin{array}{cc} Model\text{ 03 }:Teenage\;Mothers_{it}=\; & \beta_{\text{0}}+\beta_{\text{1}}Female\;Literacy_{it}+\beta_{\text{2}}Secondary\;Enrollment\;Female_{it}\\ & \;\;+\beta_{\text{3}}Poverty\;Headcount_{it}+\beta_{\text{4}}InternetUse_{it}\\ & \;\;+\beta_{\text{5}}Health\;Expenditure\;GDP_{it}+\beta_{\text{6}}Female\;Parliament_{it}+\alpha_{i}+\varepsilon_{it} \end{array}$$

$$\begin{array}{cc} Model\text{ 04}:\;Teenage\;Mothers_{it}=\; & \beta_{\text{0}}+\beta_{\text{1}}Female\;Literacy_{it}+\beta_{\text{2}}Secondary\;Enrollment\;Female_{it}\\ & \;\;+\beta_{\text{3}}Poverty\;Headcount_{it}+\beta_{\text{4}}Internet\;Use_{it}\\ & \;\;+\beta_{\text{5}}Health\;Expenditure\;GDP_{it}+\beta_{\text{6}}Female\;Parliament_{it}+u_{i}+\varepsilon_{it} \end{array}$$

The Hausman specification test was employed to determine the appropriate estimator. The test strongly supported the FE model (p < 0.01), suggesting a correlation between covariates and unit-specific effects [29]. Furthermore, the Breusch–Pagan Lagrange Multiplier (LM) test confirmed the necessity of a panel data structure by testing variance across panels [30]. To address heteroskedasticity and serial correlation in the idiosyncratic errors, robust standard errors were clustered at the panel level [31]. Two model specifications were estimated for each outcome. The baseline model included essential socio-economic and demographic controls, while the extended model incorporated additional variables related to health infrastructure, labor force participation, and adolescent-specific services to account for potential omitted variable bias. Collinearity diagnostics using the Variance Inflation Factor (VIF) shown in Table 7 indicated high collinearity among female literacy variables, prompting sensitivity analyses through stepwise exclusion and model re-estimation [32].

**Table 7:** Multicollinearity Diagnosis through Variance Inflation Factor (VIF) Results.

Independent Variable	VIF (Adolescent Fertility Model)	VIF (Teenage Mothers Model)
**Female Literacy (%)**	11.15	17.31
**Female Secondary Enrollment (%)**	7.22	6.57
**Child Stunting (%)**	5.68	7.86
**Health Exp. per capita (US$)**	5.54	7.74
**Health Expenditure (% of GDP)**	4.29	6.74
**Internet Usage (%)**	3.81	5.07
**Female Labor Force (%)**	2.29	3.34
**Physicians per 1,000**	2.20	2.96
**Urban Growth (%)**	2.19	2.18
**Female Unemployment (%)**	1.88	2.67
**Female Parliament (%)**	1.77	2.88
**Poverty Headcount (%)**	1.67	1.74
**Mean VIF**	**4.14**	**5.59**

### Spatial analysis and econometric modeling

A joint Spatial Durbin Model (SDM) has been employed in this study to estimate AFR and TMM together across 500 + districts in South Asia. It incorporated not only spatial lags but also spatially lagged covariates to uncover both direct and indirect effects. It followed the following equation:


[AFRTMM]=ρW[AFRTMM]+Xβ+WXθ+ϵ

In the above model, W stands for the spatial weights matrix that represents geographic contiguity among districts, and ρ stands for the spatial lag that indicates the influence of fertility or motherhood outcomes in one district on those in neighboring districts. The matrix X represented key independent variables like female literacy, internet use, child stunting, and contraceptive prevalence, while β estimated their direct strength of effects. The spatial spillover effects from neighboring districts and the normally distributed error term were denoted by the term WXθ and ϵ, respectively. Through this approach, the model captured both localized drivers as well as spatial dependencies, presenting a better understanding of reproductive health outcomes in a geographically interconnected region. Due to its capability of capturing both endogenous interaction effects and exogenous spillovers, the SDM has been widely used in spatial econometrics [28,33,34]. To date, it is known that this study will be the first spatially separated, subnational analysis of adolescent fertility and teenage motherhood across South Asia, utilizing spatial econometric tools. Through merging both outcomes in a model and identifying direct and spillover effects, the study can help policymakers devise targeted interventions in vulnerable districts and across shared borders.

Recognizing that policy outcomes and socio-economic behaviors in one administrative unit may affect neighboring units, the second analytical layer applied spatial econometric techniques. Specifically, the Spatial Durbin Model (SDM) was utilized as it accounts for both endogenous interaction effects and exogenous spatial lags of the regressors. The models are defined as:


$$\begin{array}{c} y=\rho Wy+X\beta +WX\theta +\epsilon ,\;\epsilon \sim N\left(\text{0},\sigma^{\text{2}}I\right) \end{array}$$

$$\begin{array}{c} y=X\beta +u,\;u=\lambda Wu+\epsilon \end{array}$$

$$\begin{array}{c} y=\rho Wy+X\beta +WX\theta +\epsilon \;\epsilon \sim N\left(\text{0},\sigma^{\text{2}I}\right) \end{array}$$

In this equation, *y* is the dependent variable vector across regions, *ρ* denotes the spatial autoregressive coefficient capturing interdependence in outcomes, *W* is the spatial weights matrix reflecting contiguity or economic proximity, *X* is the matrix of explanatory variables, *ß* is the coefficient vector, *Ɵ* measures spillover effects, and *ϵ* is the error term assumed to be normally distributed [35,36]. The spatial weights matrix *W* was constructed using both Queen contiguity (first-order adjacency) and an inverse-distance-based economic similarity index to capture multiple spatial interaction structures. Spatial autocorrelation in the dependent variables was assessed using Global Moran’s I and Local Indicators of Spatial Association (LISA), both of which indicated statistically significant spatial clustering [19]. Model estimation employed maximum likelihood techniques with heteroskedasticity-consistent standard errors [37]. The interpretation of spatial models requires decomposing the estimated effects into direct effects (the impact of a unit’s characteristics) and indirect effects (influences from neighboring units). This was achieved through the matrix transformation of the reduced form impact measures such as:


$$\begin{array}{c} \frac{\partial y}{\partial x_{r}}=(I-\rho W{)}^{-\text{1}}\left(I\beta_{r}+W\theta_{r}\right) \end{array}$$

Such decomposition allows policymakers to understand not only how local changes in predictors influence local outcomes but also how those changes diffuse spatially across regions [41].

In research, addressing potential endogeneity is crucial, especially reverse causality in variables. To mitigate this problem, lagged explanatory variables were introduced, and instrumental variable techniques were considered where theoretical justifications allowed [38]. Model robustness and interpretability were further tested through alternative spatial weight specifications and re-estimation on sub-samples.

### Justification of methodology

To make sure the study is reliable, valid and rigorous, several steps were followed. At first, the variables and methods of this study were verified by experts in public health and spatial statistics. Then, the highly correlated variables were either simplified or removed to avoid abnormal results. After that, the models were randomly checked using data from different periods to test whether the results remained stable. The study also examined the sensitivity of the models to changes in spatial assumptions using different ways to define how areas are linked. To minimize potential bias in some predictors, the analysis included some additional variables that satisfied earlier conditions. Lastly, the Gini index estimated inequality in the outcomes of reproduction, which presented unfair allocations across the territories. These attempts increased the credibility alongside the uniformity of the results.

### Ethical strength of the study

Since the research employed publicly available data with no personal or private information, there was no need to formally obtain approval for ethics. But the research followed proper and ethically suitable research procedures. Geographic boundaries were properly defined using internationally compatible procedures of avoidance of bias. Data preparation procedures were clearly defined by the research team from the data cleaning phases until report preparation. These actions certified that the study strongly considered ethical standards and warmly promoted openness, fairness, and academic honesty.

## Results

Table 2 demonstrates the descriptive statistics for all study variables collected from eight South Asian countries from 1990 to 2023. Though the average Adolescent Fertility Rate (AFR) was 72.14 births per 1,000 adolescent girls aged 15–19, a very high standard deviation of 47.29 births marked the significant variation, containing relatively low adolescent birth rates in some areas, while others suffered from alarmingly high rates. In Fig 1, a spatial map demonstrated AFR in 2023 across seven southeast Asian countries.

This map shows the geographic variation in AFR across the study regions. The color gradient represents AFR levels, ranging from 10–20, 20–30, 30–40, and ≥40 births per 1,000 women aged 15–19, where lighter shades indicate lower fertility rates and darker shades indicate higher fertility rates. These categories highlight regional disparities and identify areas with elevated adolescent fertility.

The Teenage Mothers’ (TMM) prevalence was even more diversified, with an average of more than 131.81 births per 1000 teenage girls along with a very high standard deviation of 96.89, which indicates wide variations in teenage motherhood across regions. Since female secondary enrollment and female literacy are closely related, they had very close statistics, with averages of 53.09% (SD = 27.27%) and 52.85% (SD = 27.48%), respectively. As a representation of digital access, this study finds that only 13.28% of women use the internet, which was generally very low, with a median of only 3.87%. In Table 3, country-specific statistics further emphasized these contrasts with study indicators. Holding the highest adolescent fertility of 123.5 births per 1,000 live births and teenage motherhood of 273, Bangladesh, for instance, demonstrates continuing challenges in adolescent reproductive health. However, Sri Lanka not only holds the lowest adolescent fertility rate of 23.1 consistently, but also possesses higher education levels, like female secondary enrollment reaching 93.6%. Meanwhile, countries like Afghanistan and Pakistan lag with secondary enrollment rates below 25%. This striking country-level heterogeneity underscores the necessity for disaggregated analysis, as national averages alone hide critical local differences.

While not shown graphically here, visual examination of time trends was carried out on major variables during the study period. Generally, there tends to be a declining trend in both adolescent fertility and teenage motherhood rates in the majority of the nations of South Asia, the speed and magnitude of those declines varied significantly between nations. For instance, the Maldives and Sri Lanka demonstrated a steeper and sustained fall in adolescent fertility, Afghanistan and Bangladesh tended towards slower declines or fluctuations. Correspondingly, the trend in Female Secondary Enrollment and Internet Usage demonstrated steady improvements, particularly from around the early 2000s. The regression analysis set out to determine the drivers of adolescent fertility and rates of teenage motherhood based on panel data models. Two models were estimated for each of the dependent variables: a parsimonious model (Model 01/02_01) and an augmented model with additional controls (Model 01/02_02). The Hausman test results significantly favored Fixed Effects (FE) models vis-à-vis Random Effects (RE) models, where p-values below 0.001 were obtained in all models. From the FE models, a high proportion of variation within regions across time was explained. For adolescent fertility, R² (Within) was high at 0.807, translating to about 81% of the alterations in adolescent rates of fertility within regions within the model variables. For teenage motherhood, explanatory power was low (R² Within ≈ 0.194).

One of the strongest and most consistent findings was the negative association between female literacy and adolescent fertility. Specifically, the regression coefficient (β = −1.297, p < 0.01) implies that for every one percentage point increase in female literacy rates, adolescent fertility declines by approximately 1.3 births per 1,000 adolescent girls.

Similarly, female secondary enrollment had a statistically significant negative relationship with adolescent fertility (β = −0.596, p < 0.01). This suggests that a one percentage point increase in the proportion of girls enrolled in secondary school corresponds to a reduction of about 0.6 births per 1,000 adolescent girls. Secondary schooling generally gives girls the needed life skills, social network, and higher aspirations, which can delay marriage and childbirth. It can also teach family planning and reproductive health knowledge. This finding reinforces the importance of investment in not only basic literacy but also higher education of girls in achieving meaningful reductions in adolescent fertility.

Adolescent fertility, a proxy of adolescent pregnancy, was associated significantly and positively associated with child stunting (β = 0.495, p < 0.05). This amounts to a one percentage-point increase in the proportion of child stunting in a region, translating into a 0.5 increase per 1,000 adolescent girls in adolescent fertility.

Health expenditure per capita also emerged as an important factor. Although the coefficient (β = −0.068, p < 0.01) appears small, it indicates that increased investment in health services is associated with lower adolescent fertility rates. Higher health spending likely improves the availability and quality of reproductive health services, including contraception access, counseling, and maternal care.

The teen motherhood model had some of the same relationships but generally weaker explanatory power. For example, female literacy and secondary school enrollment were, on average, negatively related to teen motherhood but not quite so strongly or reliably as adolescent fertility. Interestingly, female unemployment was positively related to teen motherhood (β = 4.741, p < 0.10), which would suggest economic desperation and lack of work opportunities may lead to early motherhood among female adolescents.

## Discussion

This study found female empowerment, which was measured through female literacy rates and secondary school enrollment, to be a prominent determinant that demonstrated a strong and statistically significant negative association with adolescent fertility as well as teenage motherhood. This finding is entirely in sync with a large body of widespread literature emphasizing female schooling’s significance in decreasing teen pregnancy [39–42]. Education empowers women by promoting awareness, hastening their knowledge on reproductive health, bolstering decision-making power, and increasing their employment prospects. On the other hand, child stunting, serving as an indicator of acute malnutrition and long-term poverty, was found to be positively and significantly correlated with higher adolescent fertility rates. This suggests that poor nutritional status is closely linked with early childbearing within the communities. Evidence that child stunting, rooted in entrenched poverty, acts as a driver rather than a predictor underscores the need for models that capture cyclical feedback loops between poverty, health, and education, rather than linear causality. Additionally, this study also found that limited public health budgets and higher unemployment rates among females were associated with increased adolescent fertility rates and teenage motherhood. The relationship between female unemployment and teenage motherhood identified here contrasts somewhat with other studies, which have found inconsistent effects of employment on adolescent pregnancy [43]. Similarly, various systematic reviews and studies identified that women’s involvement in income-generating activities has a noticeable positive effect on socioeconomic status, which has a direct influence on delaying childhood pregnancies [44,45]. However, this discrepancy may arise from regional economic differences, perhaps the availability of employment opportunities, or cultural diversity, which drives the variation of effects employment has on reproductive choices, indicating a need for more precise, context-specific investigation.

The second meaningful finding of our analysis is adolescent fertility’s strong positive association with child stunting, revealing a broader, cycle-oriented pattern of poverty and health difficulties. Serious physical and cognitive development impairment due to acute malnutrition during early childhood can obstruct educational outcomes and access to economic opportunities; hence, early marriage and motherhood serve as survival strategies within disadvantaged populations. Health spending is a weaker predictor than education and stunting but had a statistically significant negative impact on teenage fertility. This implies that increased spending in public health raises access to family planning facilities such as contraception, antenatal care, and pre-marital counseling, which are central in the prevention of adolescent or unwanted pregnancies.

Our spatial econometric analysis further shows that these determinants extend beyond local contexts, diffusing across regions through cultural and economic ties. This highlights the importance of incorporating spatial interdependencies and social networks into theoretical models of adolescent fertility to more accurately reflect how disadvantage is transmitted and sustained.

The results hold immediate implications for program and policy planning. Expanding access to education not only delays early marriage and childbearing but also builds skills and agency, thus overcoming one of the critical structural causes of adolescent childbearing. The observed positive association between child stunting and early childbearing highlights the value of integrative strategies that combine reproductive health with maternal and child nutrition programs. In the same vein, the negative effect of lockdown on adolescent fertility means more budgetary inputs toward adolescent-friendly reproductive health services are required, particularly in those areas that are “contraceptive deserts.” Increased access to reproductive health care, counseling, and contraception can go a long way in lessening the risk of early pregnancy. Combating female unemployment by way of vocational training and economic empowerment programs also becomes the key. Economic insecurity can lead some young women into early motherhood, while exposure to work and livelihood options can provide alternative life paths. By concentrating interventions on high-risk clusters that have been mapped out, policymakers can utilize resources in a better and more effective manner.

## Conclusion

This study finds that adolescent fertility and teen motherhood in South Asia and Bangladesh are not evenly spread or distributed but cluster at specific geographical hotspots, which can be otherwise known as “hotspots” of low contraceptive coverage and developmental deprivation. These hotspots identify regions reporting significantly higher rates of pregnancy among teenagers compared to other regions. Our panel regression evidence confirms that higher female literacy and more secondary school attendance are always correlated with lower adolescent fertility levels. Poverty proxies like child stunting, on the other hand, are inversely linked with fertility, which is a testament to the poverty trap. Additionally, we see that the determinants of adolescent fertility rates are different from those of teenage motherhood prevalence. For example, teenage motherhood prevalence is negatively related to child stunting, implying that the two measures must be treated separately in policy making. Overall, these results underscore strong spatial and socioeconomic inequalities that are persistent over time and warrant targeted intervention.

These results make a useful contribution to the widespread demographic and reproductive health theories by showing that the spatial context matters. Traditional models with the assumption that populations exist as homogeneous units, ignoring spatial heterogeneity, could neglect underlying dynamics that condition fertility behavior. Our evidence has implications for grounding spatial dependency in theories since geography is ignored at the expense of producing misleading conclusions about what drives adolescent fertility. By distinguishing adolescent fertility as a rate (how often it happens) from teen motherhood as a prevalence (how many girls are mothers), this study refines demographic ideas and models. The varying impact of child stunting on both of these outcomes also reflects the role of mortality and malnutrition and suggests that theoretical models must take into account complex health and social interactions on adolescent reproductive outcomes.

### Policy recommendations

For policymakers and health practitioners, these findings suggest that across-the-board programs at the national level would be ineffective in tackling teen motherhood and youth fertility. Instead, geographically focused interventions are needed to target available resources on the most trouble-prone areas, such as along the India-Bangladesh and Nepal-India borders, which have been identified as high-risk areas. The high, inverse relationship between teen fertility and female education highlights the prime importance of pushing girls’ secondary school completion and access as a sound instrument in preventing early pregnancy. The surprising, positive relationship between internet use and fertility rates also warns that it is not enough to only widen internet access. Policy makers must incorporate technology into culturally appropriate reproductive health education and digital literacy so that young women are able to benefit fully from the new sources of information and can be empowered to make decisions.

### Limitations

Certain limitations in this research should be mentioned. First, as it is the analysis based on national and sub-national aggregate data, ecological fallacy, i.e., the pattern observed at the group level may not apply to individuals, is possible. Second, multicollinearity between female literacy and school enrollment is possible, which does not allow to separately isolate their distinct impact, although this was statistically controlled to the extent possible. Third, the analysis could not account for some of the most important factors, such as the quality and availability of contraceptive services, access to safe abortion, and basic cultural attitudes toward marriage and childbearing, since there were no suitable quantitative data available. This means that the findings must be interpreted cautiously and suggest directions for more detailed research.

Subsequent studies would have to obtain and analyze more individual-level data with precise geographic coordinates, such as from the Demographic and Health Surveys (DHS). This would prevent ecological fallacy and provide stronger evidence of how adolescent motherhood and fertility are influenced by spatial conditions. Using advanced spatial statistical techniques like Geographically Weighted Regression (GWR) will be able to show how the effect of certain variables, like the usage of the internet varies from place to place. Moreover, combining quantitative research with qualitative methods like interviews and focus group discussions in hotspot areas would be able to provide richer insights into adolescent pregnancy trends’ social and cultural determinants, so that more effective interventions can be done.

### Declaration

During the preparation of this work the author(s) used Artificial Intelligence (AI) driven technology to improve language quality. After using this tool/service, the author(s) reviewed and edited the content as needed and will take(s) full responsibility for the content of the publication.

## Supporting information

S1 Data Which was extracted from World Bank Development Indicator ranges from 1990 to 2021, and this study employed this dataset to analyze Adolescent Fertility Rate and Teenage Motherhood in eight south-Asian countries. There is also a Figure demonstrating spatial distribution of Adolescent Fertility Rate (AFR) across the study areas.
(XLSX)
