# Supplementary material for: Spatiotemporal analysis of contraceptive deserts and adolescent fertility clusters in South Asia: A spatial econometric approach to breaking intergenerational poverty cycles
Source: PLOS Glob Public Health. 2026 Jun 11;6(6):e0005233. doi: 10.1371/journal.pgph.0005233 (PMC13258016; doi:10.1371/journal.pgph.0005233)
Supplement: Attachment — Submitted filename: Review Rebuttal_AP.docx [file pgph.0005233.s002.docx]

The reviewer is giving **constructive, section-specific expectations** rather than rejecting your work.

Reviewer #1:

Review: Methodology: The Dual spatial model seems to disappear and give way to a combination of panel data analysis and spatial econometric methods? Some clarification is needed there.

Answer: Thank you for this insightful comment. We apologize for the lack of clarity. In the revised manuscript, we clarify that the “dual-outcome spatial econometric model” refers to a modeling strategy in which two closely related outcomes—adolescent fertility and teenage motherhood—are examined jointly within a panel data framework while accounting for spatial dependence. The model does not disappear; rather, it is operationalized through panel spatial econometric specifications applied separately to each outcome. We have revised the methodology section to explicitly explain how panel data analysis and spatial econometric methods together constitute the dual-outcome modeling approach.

Review: Results: Several statements don't seem to belong to that section. They seem more appropriate for the discussion section. I expect more narrative and outline of results e.g. 308-310, 334-337.

Answer: Thank you for this helpful comment. We have carefully revised the Results section to ensure that it focuses strictly on reporting empirical findings. Interpretive and explanatory statements previously included in lines 308–310 and 334–337 have been relocated to the Discussion section. In addition, we have strengthened the narrative in the Results section by providing clearer descriptions of each table and outlining the key empirical patterns without interpretation.

Review: Discussion: This section is unnecessarily too long. Can it be made more concise?

Thank you for your valuable comment. We carefully reviewed the Discussion section and have revised it to improve clarity and conciseness. Redundant explanations and repetitive statements have been removed, and the key findings are now presented more succinctly while maintaining the essential interpretations and implications. We hope the revised version addresses your concern satisfactorily.
Review: Conclusion: This section is too long, and the use of examples can be revisited.

Thank you for your constructive feedback. We have revised the Conclusion section to make it more concise and focused. Unnecessary details and illustrative examples have been removed or streamlined, and the section now emphasizes the key findings and their implications more clearly. We hope this revision adequately addresses your concern.

Reviewer #2:

Major
Review 1) Some restructuring is needed with elements of the methods section being presented at the end of the introduction.

Answer: Thank you for this insightful comment. We have carefully reviewed the manuscript and restructured the relevant sections to improve clarity and logical flow. Methodological details that were previously placed at the end of the Introduction have been moved to the Methods section. The Introduction now focuses solely on background, rationale, and study objectives. We hope this revision addresses your concern.
Review 2) the manuscript is unnecessarily long, with a lot of information not routinely reported (renaming variables) and also duplication across the different methods subsections (and the introduction as previously mentioned).

Answer: Thank you for your detailed feedback. We have carefully revised the manuscript to improve clarity and conciseness. Information that is not routinely required (e.g., detailed descriptions of variable renaming) has been organized. We have also eliminated duplication across the Methods subsections and between the Introduction and Methods sections. The revised manuscript is now more focused and coherent. We hope these changes satisfactorily address your concern.

Review 3) there is no reference or use of a relevant research checklist, like STROBE.

Answer: Thank you for this important suggestion. We agree that adherence to a recognized reporting guideline strengthens methodological transparency. Accordingly, we have revised the manuscript in line with the **STROBE** checklist. Relevant items have been incorporated throughout the manuscript to ensure completeness and clarity of reporting. We trust that this revision addresses your concern.
Review 4) the paper, does not really report any useful spatial maps, which one would expect considering its aims.

Answer: Thank you for this valuable observation. We acknowledge that the inclusion of spatial maps is important given the aims of the study. In response, we have now incorporated relevant and informative spatial map in line number 333 on page 15. These additions enhance the presentation of our findings and better align the manuscript with its stated objectives. We hope this revision satisfactorily addresses your concern.
Review 5) there is no justification on the methodological choices e.g. the spatial durbin model, over simpler alternatives.

Answer: Thank you for this insightful comment. We have now strengthened the Methods section by clearly justifying our choice of the Spatial Durbin Model over simpler alternatives. Specifically, we explain its suitability in accounting for both spatial dependence in the outcome variable and spatial spillover effects of explanatory variables (page:12, line:257-257), which are central to our research objectives.
Review 6) ecological fallacy is a real concern but the authors only mention it in passing in the limitation section. are there any sensitivity analyses the authors could conduct?
Answer: Thank you for highlighting this important point. We acknowledge that ecological fallacy is a potential concern in our study, as our analyses are based on district-level aggregate data. While we did not perform formal sensitivity analyses, we have expanded the Limitations section to clearly discuss this issue and caution against drawing individual-level inferences. For future studies, we suggest conducting sensitivity analyses using multilevel models or disaggregated individual-level data to assess the robustness of district-level associations. We hope this clarification adequately addresses your concern.
Minor
Review 1) consider structuring the abstract

Answer: The first review mentioned the abstract is good. Therefore, we are not going to change it.
Review 2) more information is needed on interpolation and imputation for replicability.

Answer: Thank you for your important review. We have mentioned interpolation and imputation at page number 7 in line (137-143).
